# Diversification of behavior and postsynaptic properties by netrin-G presynaptic adhesion family proteins

**DOI:** 10.1186/s13041-016-0187-5

**Published:** 2016-01-08

**Authors:** Qi Zhang, Hiromichi Goto, Sachiko Akiyoshi-Nishimura, Pavel Prosselkov, Chie Sano, Hiroshi Matsukawa, Kunio Yaguchi, Toshiaki Nakashiba, Shigeyoshi Itohara

**Affiliations:** Laboratory for Behavioral Genetics, RIKEN Brain Science Institute, 2-1 Hirosawa, Wako, Saitama 351-0198 Japan

**Keywords:** Netrtin-G1, Netrin-G2, Molecular evolution, Cognitive diversification, GPI-protein, Postsynapse, Presynapse

## Abstract

**Background:**

Vertebrate-specific neuronal genes are expected to play a critical role in the diversification and evolution of higher brain functions. Among them, the glycosylphosphatidylinositol (GPI)-anchored netrin-G subfamily members in the UNC6/netrin family are unique in their differential expression patterns in many neuronal circuits, and differential binding ability to their cognate homologous post-synaptic receptors.

**Results:**

To gain insight into the roles of these genes in higher brain functions, we performed comprehensive behavioral batteries using netrin-G knockout mice. We found that two netrin-G paralogs that recently diverged in evolution, netrin-G1 and netrin-G2 (gene symbols: *Ntng1* and *Ntng2*, respectively), were responsible for complementary behavioral functions. Netrin-G2, but not netrin-G1, encoded demanding sensorimotor functions. Both paralogs were responsible for complex vertebrate-specific cognitive functions and fine-scale regulation of basic adaptive behaviors conserved between invertebrates and vertebrates, such as spatial reference and working memory, attention, impulsivity and anxiety etc. Remarkably, netrin-G1 and netrin-G2 encoded a genetic “division of labor” in behavioral regulation, selectively mediating different tasks or even different details of the same task. At the cellular level, netrin-G1 and netrin-G2 differentially regulated the sub-synaptic localization of their cognate receptors and differentiated the properties of postsynaptic scaffold proteins in complementary neural pathways.

**Conclusions:**

Pre-synaptic netrin-G1 and netrin-G2 diversify the complexity of vertebrate behaviors and differentially regulate post-synaptic properties. Our findings constitute the first genetic analysis of the behavioral and synaptic diversification roles of a vertebrate GPI protein and presynaptic adhesion molecule family.

## Background

Glycosylphosphatidylinositol (GPI)-linked proteins account for up to 20 % of membrane protein genes. GPI proteins anchor to lipid rafts and constrain the signaling cascades under fine spatiotemporal regulation. These proteins are mobile within the membrane, enabling fluid interactions with other molecules [1–3]. Comparative proteomics and phylogenic analyses indicate that organisms of higher hierarchical assemblage exhibit higher variations in GPI proteins with neuronal over-representation [4]. Functional studies of GPI proteins in the nervous system have focused on axon-glia interactions and their roles in neuronal survival, migration, and in axonal growth, guidance, fasciculation, and myelination [5–8]. GPI proteins are also involved in synaptogenesis, spine morphology, maturation, and collapse [7, 9, 10]. Most of the previous studies, however, were performed in vitro and the in vivo functions of GPI proteins, especially their genetic relationships with cognitive behavior functions in adult animals, have largely remained elusive [11–13].

The highly developed nervous system of vertebrates compared to invertebrates confers the ability to adapt to complex environments. During evolution, certain gene families underwent dynamic expansion, contraction, or extinction, and ~22 % of vertebrate genes are not found in invertebrates [14]. Why do vertebrates need these new genes and what new brain functions specific to vertebrates do they support? Moreover, how do vertebrate-specific genes contribute to the diversity and phenotypic complexity of animal behavior and synaptic properties? Recent work shows differential regulation of behavior by two expanded vertebrate families of postsynaptic signaling proteins [15, 16]. Presynaptic cell adhesion proteins, however, have not yet been assessed.

**Fig. 1:**
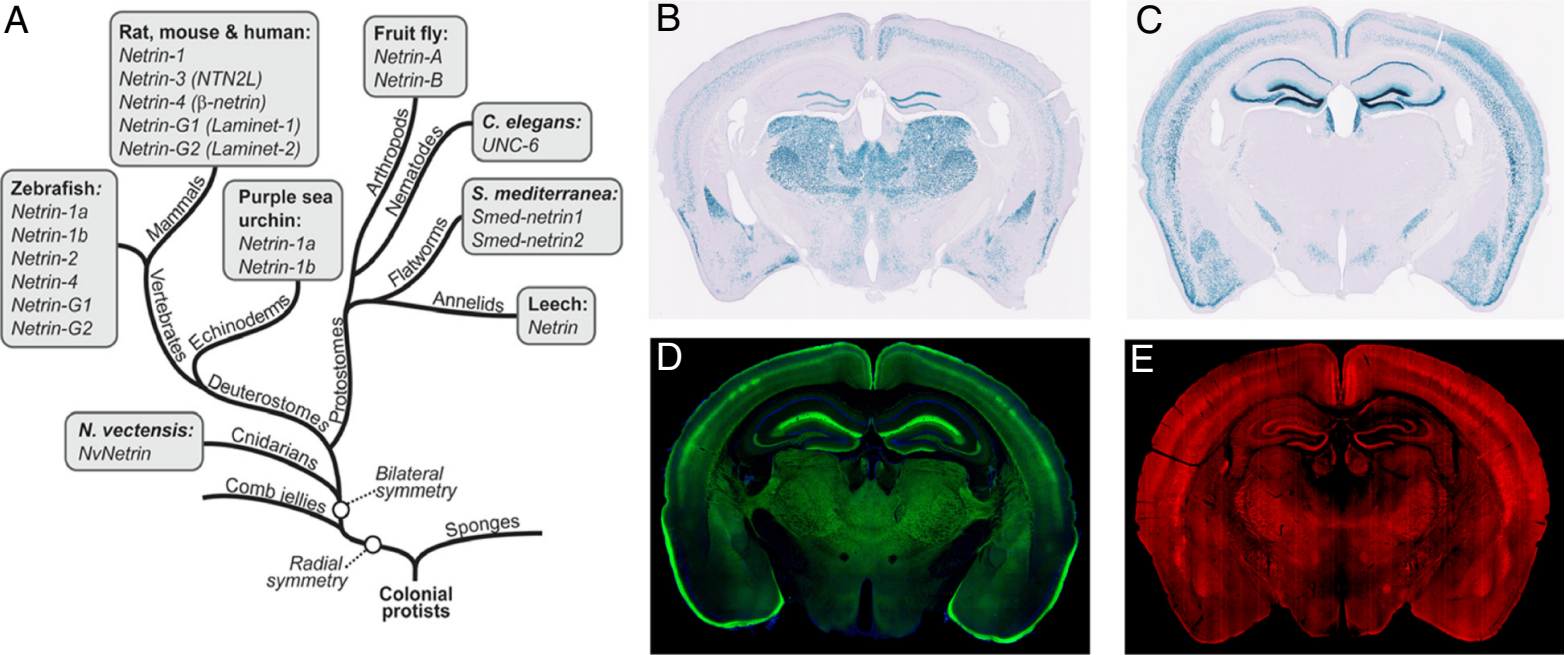
Complementary expression patterns of the two netrin-G subfamily members in the mouse brain. **a** Evolutionary tree diagram highlighting the presence of netrin homologs in a wide variety of bilaterally symmetrical organisms. Note that netrin-G family members only exist in vertebrates (With kind permission of Springer Science + Business Media, [87]. **b** and **c** Lac-Z staining of NetrinG1-NLS-lacZ-KI and NetrinG2-NLS-lacZ-KI mouse, respectively. The Lac-Z signal localizes only in the nucleus, with largely non-overlapping expression patterns of netrin-G1 and netrin-G2 genes. Images were captured by Nanozoomer (Hamamatsu photonics). **d** and **e** Brain slices from netrin-G1-Tau-EGFP and netrin-G2-Tau-mCherry mice show projections and cell bodies of distinct populations of neurons that express netrin-G1 or netrin-G2. Fluorescent images of Tau-EGFP and Tau-mCherry were captured by fluorescent microscopes (FV1000, Olympus) and (Nanozoomer, Hamamatsu photonics), respectively. The fluorescent intensity of Tau-mCherry was amplified by an indirect immunofluorescent technique using anti-mCherry antibodies

Netrin-G1 and netrin-G2 comprise a pair of GPI-anchored adhesion molecules enriched in presynaptic terminals. They are ~30 % homologous to classical netrins, but constitute an independent subfamily in the UNC-6/netrin family. Unlike classical netrins, whose orthologs exist in invertebrates to vertebrates, netrin-Gs are exclusive to vertebrates [17–19] (Fig. 1a). Null mutant mice of netrin-1 have disrupted axonal projections [20, 21]. Neither netrin-G1 nor netrin-G2, however, appears to be necessary for axon guidance [22, 23]. Strikingly, netrin-G1 and netrin-G2 are expressed in a largely complementary pattern in the adult brain (Fig. 1b-e), and bind specifically to their postsynaptic receptors NGL1 and NGL2, also known as LRRC4C and LRRC4, respectively [18, 22, 24–27]. NGL1 was originally named ‘netrin-G1 ligand’. For consistency within the UNC-6/netrin family, we use the term NGLs to represent netrin-Gs receptors. Loss of netrin-G1 or netrin-G2 disrupts the laminar distribution of their receptors [22, 23]. Abnormal expression of netrin-Gs in humans is associated with several mental disorders [28–32]. Together, these findings indicate that netrin-Gs may have isoform-specific roles and are involved in diversifying vertebrate-specific higher brain functions. Thus, the netrin-G-family provides an ideal entry point for exploring genetic animal models to understand how the evolution of GPI proteins can shape brain functions and diversify vertebrate-specific behaviors, and their complex regulation.

In this study, we performed a battery of behavioral tests using netrin-G1 and netrin-G2 knockout (KO) mice to address the roles of this pair of GPI-linked molecules in simple and complex cognitive behaviors. We also measured postsynaptic changes in netrin-G KO mice. To our knowledge, this is the first genetic dissection of the roles of a GPI protein and presynaptic adhesion molecule family in vertebrate behavior and synaptic properties.

## Results

### Netrin-G2, but not netrin-G1, encodes for high-demand sensorimotor functions

**Fig. 2:**
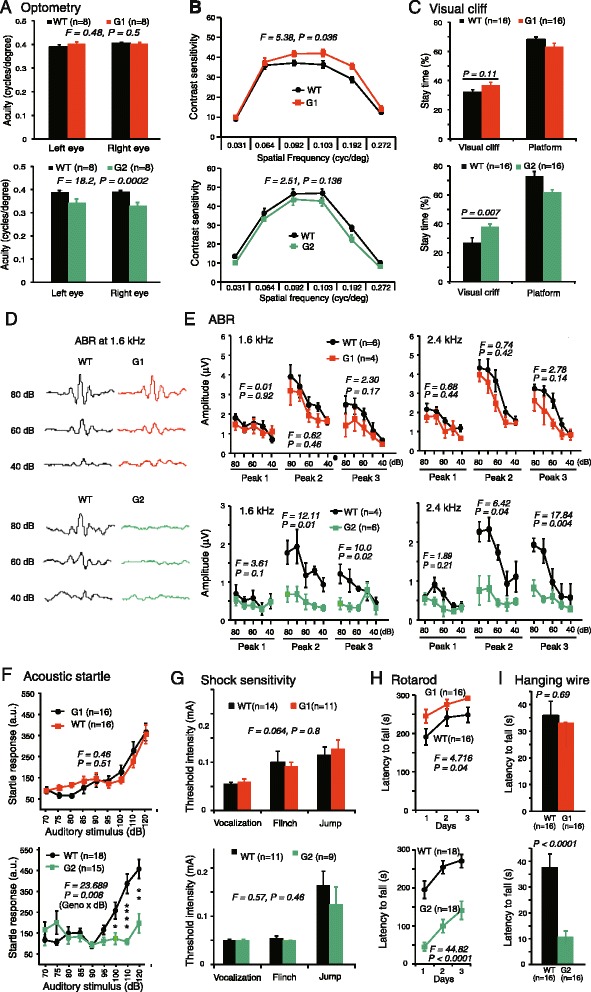
Differential phenotypes of netrin-G1 KO and netrin-G2 KO mice in sensorimotor behaviors. **a** and **b** Optometry: **a** Visual acuity in both left and right eyes was examined by measuring the highest spatial frequency the mouse could track when the grating was systematically increased. Visual acuity was comparable between netrin-G1 KO mice and WT mice [2–3 mo-old, two-way ANOVA for genotype, not significant (ns) for interaction between factors]. Netrin-G2 KO mice exhibited decreased visual acuity (4–6 mo-old, two-way ANOVA for genotype, ns for interaction). **b** Visual contrast sensitivity was evaluated by measuring the minimum contrast that could induce tracking behavior at six different spatial frequencies. Netrin-G1 KO mice showed a modest increase in contrast sensitivity (two-way ANOVA for genotype, ns for interaction). Contrast sensitivity was comparable between netrin-G2 KO mice and WT mice (two-way ANOVA for genotype, ns for interaction). **c** Visual perception of depth was examined using a visual cliff test. Netrin-G1 KO and WT mice did not differ in the time spent on the platform or on visual cliff areas (10 mo-old, Student’s *t*-test). Netrin-G2 KO mice spent more time in the visual cliff area (9 mo-old, Student’s *t*-test). **d** and **e** ABR: **d** Sample traces of the ABR. **e** Amplitude analysis of the wave peaks revealed no effect of deletion of the netrin-G1 gene (6 mo-old, two-way mixed ANOVA for genotype, ns for interaction in all analyses). In netrin-G2 KO mice, on the other hand, peaks 2 and 3 were significantly reduced (8 mo-old, two-way mixed ANOVA for genotype, ns for interaction in all analyses), while peak 1 was not affected by genotype. **f** Startle responses to auditory stimuli. Netrin-G1 KO mice did not differ from WT mice in their startle response to the auditory stimuli (3 mo-old, mixed two-way ANOVA for genotype, ns for interaction). Netrin-G2 KO mice, however, exhibited marked deficits (4 mo-old, two-way mixed ANOVA for genotype, significant interaction; * *P* < 0.05, ** *P* < 0.01, **** *P* < 0.0001, *post hoc t*-test). **g** Responses to electric foot shocks: netrin-G1 KO and netrin-G2 KO mice did not differ from WT mice in sensitivity to electric foot shocks (netrin-G1 KO, 8–9 mo-old, netrin-G2-KO, 10 mo-old; two-way ANOVA for genotype, ns for interaction in both genotypes). **h** Rotarod test: The latencies to fall off the accelerating rotarod task were compared to evaluate motor learning and coordination ability. Netrin-G1 KO remained on the rotating rod for a longer time than WT mice (2–3 mo-old; mixed two-way ANOVA for genotype, ns for interaction). Netrin-G2 KO mice remained on the rod for a shorter time (5 mo-old; two-way mixed ANOVA for genotype, ns for interaction). **i** Hanging wire test: There was no genotype difference between netrin-G1 KO and WT mice (4–5 mo-old, Student’s *t*-test). Netrin-G2 KO mice remained on the wire for a shorter time (5 mo-old, Student’s *t*-test). Data are presented as mean ± SEM

The most remarkable feature of netrin-G subfamily members of the UNC6/netrin family is their expression patterns in distinct neuronal circuits (Fig. 1). LacZ signals of knockin mice represent nuclei of cells expressing either netrin-G1 or netrin-G2 [33](Fig. 1b and c). Fluorescent proteins fused with tau polypeptides in independent knockin mouse lines represent axonal projections of these cells (Fig. 1d and e). As previously described, these mice develop normally and have no gross anatomical abnormalities [22]. An electrophysiologic study focusing on hippocampal circuits support their roles in regulating synaptic transmission and/or plasticity in a circuit specific manner [34]. To know the evolutional significance of the netrin-G subfamily members (Fig. 1a) in higher brain function, we made comprehensive behavioral tests for netrin-G1 KO and netrin-G2 KO mice. First, examination of simple neurologic reflexes, including righting, posture, eyeblink, ear twitch, and whisker orienting reflex, revealed no genotype differences (data not shown). Multiple behavioral tests were used to thoroughly examine perceptual ability and motor function. Visual acuity and contrast sensitivity of the mice were examined by measuring the optokinetic responses to a rotating sine-wave grating. Netrin-G1 KO mice exhibited comparable visual acuity (Fig. 2a, *top*) and increased contrast sensitivity compared to WT mice (Fig. 2b, *top*). Netrin-G2 KO mice, on the other hand, failed to track the grating at a higher frequency and exhibited reduced visual acuity (Fig. 2a, *bottom*). The contrast sensitivity of netrin-G2 KO mice was indistinguishable from that of WT mice (Fig. 2b, *bottom*). Visual depth perception was assayed by the visual cliff test. Similar to WT mice, both netrin-G1 KO and netrin-G2 KO mice remained in the platform region for a significantly longer time than in the visual cliff zone (*p* < 0.0001 for all groups, Student’s two-tailed *t*-test). Netrin-G2 KO mice, however, but not netrin-G1 KO mice, spent a significantly more time in the visual-cliff zone than WT mice (Fig. 2c), suggesting that their visual depth perception was impaired. Primary visual cortex is involved in detecting orientation, contrast, and binocular depth perception [35, 36]. Previous studies [37] and our unpublished data indicate that netrin-G2 and NGL2 are expressed in the visual pathway, including the retina, superior colliculus, and visual cortex, and are involved in lamination, synapse formation, and signal transmission, which may provide the molecular mechanisms underlying the visual impairment in netrin-G2 KO mice.

ABR was examined to determine auditory function (Fig. 2d, e). Amplitude analysis of the wave peaks indicated that none of the peaks examined were affected by the deletion of netrin-G1. In netrin-G2 KO mice, on the other hand, amplitudes of peaks 2 and 3 were significantly reduced. Consistent with the ABR phenotypes, netrin-G2 KO, but not netrin-G1 KO, mice exhibited deficits in auditory startle responses (Fig. 2f). Another group examined an independent netrin-G2 KO mouse line and described auditory impairment with decreased amplitude only in peak 3 [23]. This subtle discrepancy might be due to the different frequencies used for auditory stimulation or differences in the genetic backgrounds of the two independent lines. Peaks 1 to 3 of the ABR in mice are generally considered to arise approximately from the cochlear origins (peak 1), from the cochlear nucleus (peak 2), and the superior olivary complex (peak 3). Our findings suggested that the auditory deficit in netrin-G2 KO mice was caused by the neural defect originating from the cochlear nucleus or superior olivary complex, although these generators of the ABR are contested [38]. Netrin-G2 and its ligand NGL2 are abundant in the auditory circuit. In contrast to phenotypes in visual and auditory responses, no significant differences in touch sensitivity or nociceptive responses were detected between netrin-G1 KO and netrin-G2 KO mice (Fig. 2g and data not shown).

The runway task and rotarod task were examined to evaluate motor learning and coordination. The cerebellum, basal ganglia, and motor cortex are highly involved in these tasks [39, 40]. After repeated training in the runway task, both types of netrin-G KO mice exhibited a normal reduction in the latency to reach the top platform, and KO and WT mice did not significantly differ in their overall performance (data not shown). When exposed to a rotarod test, however, which is a more demanding task, netrin-G1 KO and netrin-G2 KO mice produced contrasting results. Netrin-G1 KO mice remained on the rotating rod significantly longer than WT mice, whereas netrin-G2 KO mice performed poorly (Fig. 2h). The better performance of netrin-G1 KO mice may reflect their lighter bodyweights [22]. To gain insight into the reasons for the poor performance of netrin-G2 KO mice on the rotarod test, we evaluated their performance in a hanging wire test and grip strength test. Netrin-G1KO mice and WT mice held onto the wire for a similar duration of time, while netrin-G2 KO mice held onto the wire for a significantly shorter duration of time compared to WT mice (Fig. 2i). A grip strength test was used to measure forelimb strength, and both netrin-G1 KO and netrin-G2 KO mice exhibited normal grip strength (data not shown). Thus, the impaired performance of netrin-G2 KO mice in the higher demanding motor tasks is likely due to deficits in body balance and motor coordination, and not muscle strength. In summary, a netrin-G1 gene deficit did not affect the performance of mice in sensorimotor tasks. A netrin-G2 deficit, however, impaired visual, auditory, and motor coordination abilities required for demanding tasks. We do not rule out the possibility that netrin-G2 KO mice may also have difficulties in keeping motivation to stay on the rod and wire.

### Netrin-G1 and netrin-G2 dissociate emotional behaviors

**Fig. 3:**
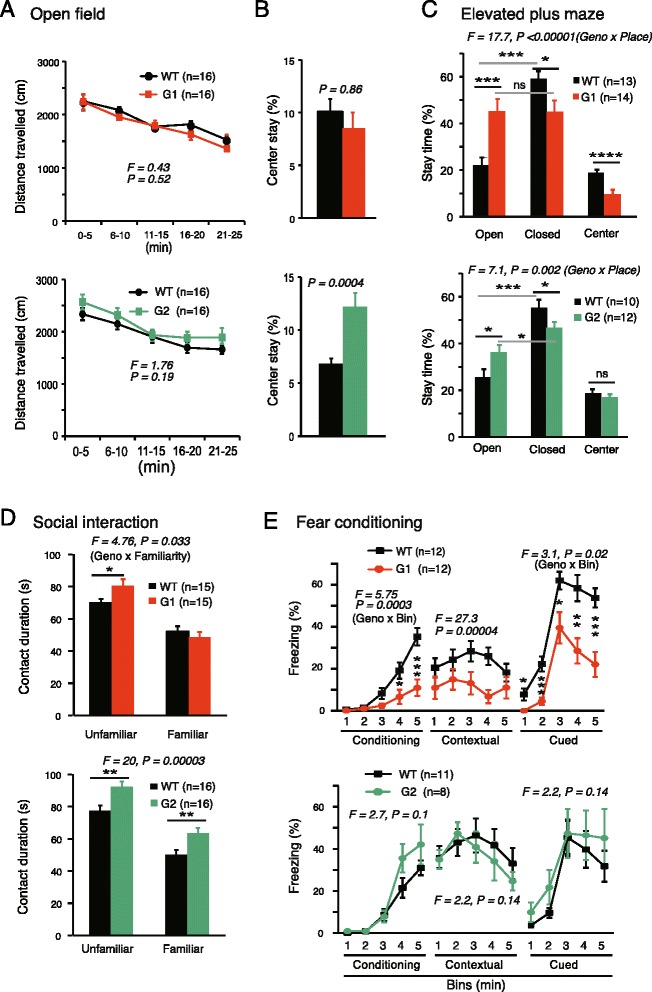
Differential phenotypes of netrin-G1 KO and netrin-G2 KO mice in the emotional domain. **a** and **b** Open field test: **a** Distance traveled by netrin-G1 KO and netrin-G2 KO mice was equivalent to that of WT mice (netrin-G1 KO, 3 mo-old; netrin-G2 KO, 3 mo-old; two-way mixed ANOVA for genotype, ns for interaction). **b** Place preference to the center over the periphery was not affected in netrin-G1 KO mice, while netrin-G2 KO mice spent more time in the center area (Student’s *t*-test). **c** Elevated plus maze test: WT mice preferred to stay in the closed arms. Netrin-G1 KO mice showed no arm preference and remained in the center area for a shorter time (6–7 mo-old, 2-way ANOVA for genotype and place, significant interaction; *post hoc t*-test, * *P* < 0.05, *** *P* < 0.001, **** *P* < 0.0001; ns, not significant). Netrin-G2 KO mice spent significantly more time in the open arms and did not differ from WT mice in the time spent in the center area (6–7 mo-old, 2-way ANOVA for genotype and place, significant interaction; *post hoc t*-test, * *P* < 0.05, *** *P* < 0.001). **d** Social recognition test: All genotypes interacted with juvenile mice for a significantly shorter time than WT mice when they encountered the same mouse on the second day. Netrin-G1 KO mice had a longer contact duration with unfamiliar but not familiar mice (3 mo-old, 2-way mixed ANOVA for genotype and familiarity, significant interaction, * < 0.05 *post hoc t*-test). Netrin-G2 KO mice had a longer contact duration with both unfamiliar and familiar mice (3 mo-old, 2-way ANOVA for genotype and familiarity, ns for interaction, ** < 0.01 *post*-*hoc t*-test). **e** Fear conditioning test: The freezing response was markedly reduced in netrin-G1 KO mice during conditioning (8 mo-old; 2-way mixed ANOVA for genotype and bin, ns for interaction, *post*-*hoc t*-test, * *P* < 0.05, *** *P* < 0.001). Netrin-G1 KO mice also showed a decreased freezing ratio in the contextual memory test (2-way mixed ANOVA for genotype, ns for interaction) and cued memory test (2-way mixed ANOVA for genotype, ns for interaction; *post*-*hoc t*-test, * *P* < 0.05, ** *P* < 0.01, *** *P* < 0.001). Netrin-G2 KO mice were not different from WT mice in any session (9–10 mo-old). Data are presented as mean ± SEM

Anxiety levels in the netrin-G KO mice were examined using the open field (OF) and elevated plus maze (EPM) tests. In the OF test, both netrin-G1 KO and netrin-G2 KO mice demonstrated normal locomotive activity (Fig. 3*a*). Compared with WT mice, the percent time spent in the center of the OF box was not different in netrin-G1 KO mice, but almost doubled in netrin-G2 KO mice (Fig. 3*b*). In the EPM test, netrin-G2 KO mice spent significantly more time the open arms than WT mice (Fig. 3*c bottom*). These results together with the data from the visual cliff test consistently suggested reduced anxiety levels of netrin-G2 KO mice. Deficits in visual perception may partially underlie the reduced anxiety. The data from netrin-G1 KO mice were unique. Netrin-G1 KO mice showed anxiolytic behaviors in the EPM, but not in the OF and visual cliff tests. The reasons for the differential anxiety-related behaviors of netrin-G1 KO mice are unclear. State-dependent fear responses may be fundamental in humans. Netrin-G paralogs might have diversified to detect and discriminate contexts and to selectively regulate different forms of anxiety and fear. It should be also noted that netrin-G1 KO mice, but not netrin-G2 KO mice, stayed in the center area of the EPM for a shorter time (Fig. 3c), reflecting reduced conflict-related behaviors in the center area. This may represent an impulsive nature of netrin-G1 KO mice, as discussed later.

The sociability of the netrin-G KO mice was assessed by investigating their interactions with unfamiliar juvenile mice in a novel environment. Exposure to the same juvenile male after a 24-h delay resulted in a significantly reduced interaction time in all genotype groups (*p* < 0.001 for all), indicating that both types of KO mice retained substantial social recognition memory (Fig. 3*d*). Netrin-G1KO mice displayed a significant increase in the total duration of physical contact during the first exposure compared with WT mice, but the interaction time eventually returned to the WT level during the second exposure (Fig. 3*d top*). The duration of physical contact was longer for netrin-G2 KO mice during both the first and second exposures (Fig. 3*d bottom*). Mice with lesions in the prelimbic area of prefrontal cortex are not able to disengage from an action leading to some kind of reward (social partner) and time spent in social contact is enhanced [41]. Netrin-G1 and netrin-G2 distribute in different layers of the prelimbic cortex and may provide a new entry point to address the layer-specific contribution to social behaviors.

Emotional responses such as fear are expressed as freezing in rodents and are widely used for assessing context-dependent and cue-dependent association memory. Many studies have demonstrated that the hippocampus and amygdala are involved in context-dependent fear memory, and the amygdala is indispensable for cue-dependent fear memory [42]. Netrin-G1 and netrin-G2 are abundantly expressed in distinct circuits in these brain areas. Netrin-G1 KO mice exhibited significant reductions in immediate freezing responses during conditioning, and freezing responses in the 24-h contextual and 48 h cue-dependent memory tests (Fig. 3e*top*). Considering that netrin-G1 KO mice exhibited no differences in sensitivity to electric foot shocks (Fig. 2g), these data suggest that netrin-G1 KO mice have deficits in encoding fear responses. In contrast, the freezing responses of netrin-G2 mice did not appear to differ at any stage of the fear-conditioning test (Fig. 3e*bottom*). Note that the conditional stimulus we used was white noise, which covered a broad range of frequencies. Evidently the residual auditory information and/or somatosensory signals evoked by tone were sufficient to encode and retrieve the fear memories in netrin-G2 KO mice.

Taken together, these findings indicate differential characteristics of netrin-G1 KO and netrin-G2 KO mice in the emotional domain.

### Netrin-G1 and netrin-G2 dissociate complex cognitive behaviors

**Fig. 4:**
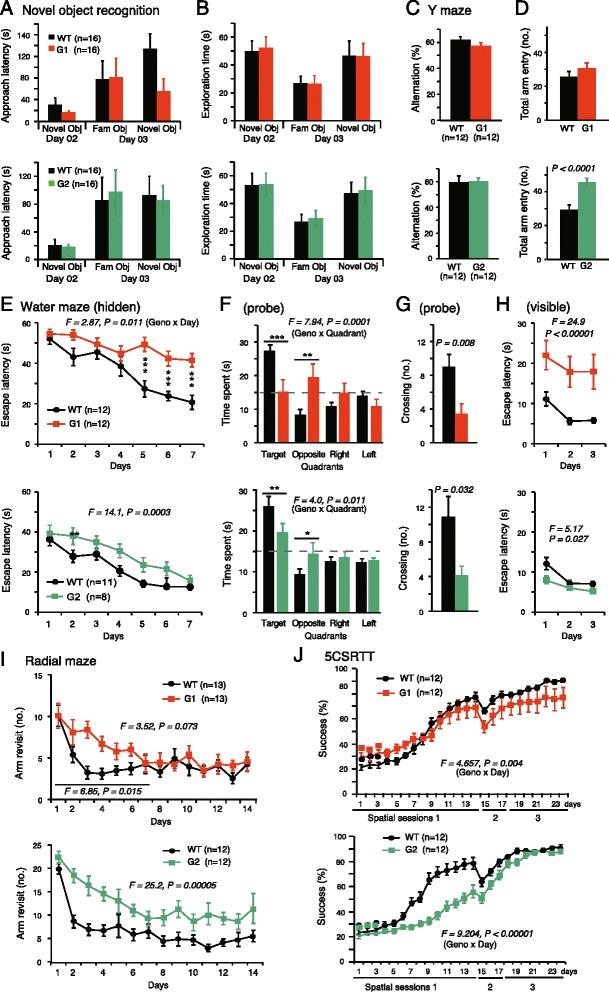
Differential phenotypes of netrin-G1 KO and netrin-G2 KO mice in learning and memory. **a** and **b** Object recognition test: **a** Latencies of netrin-G1-KO (3 mo-old) and netrin-G2 KO (3 mo-old) mice to approach novel and familiar objects were not significantly different. Mice were exposed to the open field on Day 1 (see Fig. 3). **b** Comparable to WT mice, both netrin-G1 KO and netrin-G2 KO mice spent a significantly greater percentage of time exploring the novel object than the familiar object during Day 3 (paired *t*-test: *p* < 0.01 for all groups). No genotype differences were detected. **c** and **d** Y-maze task: **c** Percent arm alternations in all groups was above the chance level (netrin-G1 KO, 13 mo-old; netrin-G2 KO, 13 mo-old). No genotype differences correlated with the percentage of spontaneous arm alterations. **d** Netrin-G2 KO mice exhibited a significant increase in the number of arm entries (Student’s *t*-test). **e**-**h** Spatial reference memory was examined using the Morris water maze task: **e** Netrin-G1 KO mice had a longer latency than WT mice to reach the hidden platform (6–7 mo-old; 2-way mixed ANOVA for genotype and day, ns for interaction; ***p < 0.001 *post*-*hoc t*-test). Netrin-G2 KO mice also had a longer latency (9–10 mo-old; 2-way mixed ANOVA for genotype, ns for interaction). **f** In the probe test, netrin-G1 KO and netrin-G2 KO mice spent significantly less time spent in the target quadrant (2-way ANOVA for genotype, ns for interaction, *P < 0.05, **P < 0.01, and *** < 0.001 *post*-*hoc* Bonferroni test). **g** Both netrin-G1 KO and netrin-G2 KO mice had significantly fewer crossings over the previous platform site (Student’s *t*-test). **h** The latency to reach the platform in the visible platform session was also significantly prolonged in netrin-G1 KO mice (2-way mixed ANOVA for genotype, ns for interaction). Netrin-G2 KO mice had a modestly shorter latency (2-way mixed ANOVA for genotype, ns for interaction). **i** Arm revisit errors were recorded in the radial maze task: netrin-G1 KO mice made a significantly greater number of errors in the early stage of training (3–4 mo-old; 2-way mixed ANOVA for genotype, ns for interaction). Netrin-G2 KO mice made significantly more revisit errors than WT mice in the all stages (4 mo-old; 2-way mixed ANOVA for genotype, ns for interaction) **j**: 5CSRTT was used to examine learning acquisition and spatial attention abilities: For netrin-G1 KO mice, the success rate was comparable between genotypes in session 1, and gradually and slightly declined in later sessions (7 mo-old; 2-way mixed ANOVA for genotype, ns for interaction). Netrin-G2 KO mice showed a significant delay in learning the task (7 mo-old; 2-way mixed ANOVA for genotype, ns for interaction). Data are presented as mean ± SEM

A wide range of cognitive tasks was used to evaluate the learning and memory ability of netrin-G KO mice. In simple span tasks, both netrin-G1 KO and netrin-G2 KO mice behaved similarly to their WT littermates. For example, similar to WT mice, netrin-G1 KO and netrin-G2 KO mice exhibited an exploratory preference toward novel over familiar objects in the object recognition test (Fig. 4*a*, *b*). In the Y-maze spontaneous alternation test, both types of KO mice tended to enter the alternate arm, similar to WT controls (Fig. 4*c*). Netrin-G2 KO mice, but not netrin-G1 KO mice, had a significantly greater number of arm entries compared to WT controls, indicating the hyperactive locomotion of netrin-G2 KO mice under this condition (Fig. 4*d*).

Complex span tasks, including the Morris water maze, radial maze, and 5CSRTT, were applied to examine the roles of the netrin-Gs in higher cognitive functions. The Morris water maze test is widely used to analyze hippocampus-dependent spatial learning and memory [43]. In the hidden platform test, netrin-G1 KO mice required significantly more time to reach the platform (Fig. 4*e top*). Netrin-G1 KO mice spent significantly less time in the target quadrant and had significantly fewer crossings over the previous platform site in the probe test (Fig. 4*f*, *g top*). They also had prolonged latencies to reach the platform in the visible platform test (Fig. 4*h top*). Because netrin-G1 KO mice performed well in all visual ability and motor coordination tests, we further tested their motivation in the forced swimming task. No difference in the floating time was detected between KO and WT mice (data not shown). Interestingly, the deficits of the netrin-G1 KO mice in the hidden test and probe test were largely attenuated when the visible test was performed first for another experimental group, although they had a slightly slower swim speed in the tests (data not shown). Together, these findings suggest that netrin-G1 KO mice have deficits in processes requiring perception of a goal-oriented strategy, but not for spatial reference memory. Netrin-G2 KO mice swam normally. In the hidden platform test, netrin-G2 KO mice had an increased latency to reach the platform (Fig. 4e*bottom*). In the probe test, netrin-G2 KO mice swam in the target quadrant for a shorter time compared to WT mice (Fig. 4*f bottom*), and crossed the previous platform site less frequently (Fig. 4*g bottom*), indicating inaccurate spatial memory. The latency to reach the visible platform was not affected by genotype (Fig. 4*h bottom*), suggesting that motivation, vision, and motor coordination of the mutant mice were sufficient to accomplish this task. These findings suggest that netrin-G2 KO mice had a spatial learning and memory deficit.

Spatial working memory was examined in the 8-arm radial maze test, in which success is reportedly dependent on hippocampal and prefrontal cortex function [44–47]. Omission errors were equivalent among the three groups, indicating that both netrin-G1 KO mice and netrin-G2 KO mice exhibited normal adaptation to the experimental apparatus and sufficient sensorimotor ability to accomplish the task. The performance curves of the netrin-G1 KO mice indicated a remarkable deficit in learning progression, i.e., significantly more errors were observed during the early stage (first week) of the training, but not during the later stage (second week; Fig. 4*i top*). Netrin-G2 KO mice made significantly more revisit errors than WT mice during the entire 2-week process (Fig. 4*i bottom*), indicating that netrin-G2 has a crucial role in the neuronal circuits involved in spatial working memory. We used two different arms, high wall and low wall arms, for netrin-G1 KO and netrin-G2 KO mice, respectively. With the low wall arms, netrin-G1 KO mice tended to climb up the walls and jump off of the arm, perhaps reflecting impulsivity.

In the early spatial sessions (spatial sessions 1–3) of the 5CSRTT, mice must first learn to link a nose-poke into the illuminated single hole among five holes with a reward. The success rate of netrin-G1 KO mice was indistinguishable from that of WT mice (Fig. 4*j top*). Netrin-G2 KO mice, however, exhibited a marked deficit during spatial session 1 (Fig. 4*j bottom*). Netrin-G2 KO mice improved more slowly, indicating a deficit in procedural learning, but eventually reached the level of WT mice.

Taken together, these findings indicate that netrin-G1 and netrin-G2 have differential roles in the neuronal circuits involved in various demanding cognitive functions, and that netrin-G2-dependent circuits have a larger impact on various forms of learning and memory.

### Netrin-G1 and netrin-G2 affect attention behaviors

Attention is a fundamental neuronal mechanism underlying demanding cognitive functions and attention deficits are associated with various psychiatric disorders associated with single nucleotide polymorphisms in netrin-G1 and netrin-G2 genes [28–32]. Moreover, brain areas involved in attention control, such as the prefrontal cortex, anterior cingulate gyrus, parietal and posterior cortex, thalamus and superior colliculus [48], largely overlap with the circuits expressing either netrin-G1 or netrin-G2 [33]. Attention processes can be selective or non-selective [49]. The non-selective type is represented by rearing episodes (vertical activity) in rodents [49].

**Fig. 5:**
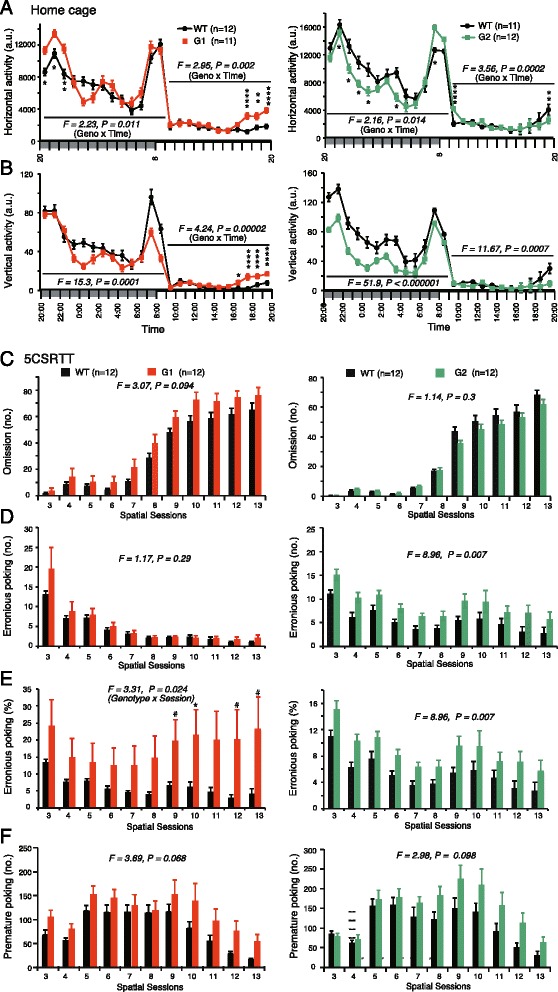
Differential phenotypes of netrin-G1 KO and netrin-G2 KO mice in attention behaviors. **a** and **b** Home cage activity (netrin-G1 KO, 6-mo old, netrin-G2 KO, 3 mo-old): All mice were entrained under a 12-h dark and 12-h light cycle and their locomotor activities were recorded over 7 days. The circadian rhythm of the netrin-G KO mice did not appear to be grossly deviant from that of the WT mice. Average amounts of both horizontal (**a**) and vertical activity (**b**) over 6 days (days 2–7) were calculated in 1-h time bins for all genotypes (a.u. represents arbitrary unit; 2-way mixed ANOVA). Netrin-G1 KO mice were characterized by less vertical activity, representing attention behavior during the active phase, and hyperactivity during the late resting phase, indicating alterations in sleep. Netrin-G2 KO mice showed significantly decreased vertical activity, suggesting alterations in attention. **c**-**f** Attention-related behaviors were assessed by 5CSRTT (netrin-G1-KO and netrin-G2 KO, 7 mo-old at the beginning): **c** Netrin-G1 KO mice tended to make omission errors although there was no statistical significance (2-way mixed ANOVA for genotype, ns for interaction). **d** Netrin-G2, but not netrin-G1, KO mice exhibited increases in erroneous nose-poke numbers throughout the sessions (2-way mixed ANOVA for genotype, ns for interaction), indicating attention deficits. **e** Netrin-G1 KO mice exhibited higher erroneous nose-poke rates per response in later sessions (2-way mixed ANOVA for genotype, ns for interaction, *post*-*hoc t*-test). **f** Both genotypes tended to show premature responses, reflecting impulsivity, more frequently than WT mice although there was no overall statistical significance (2-way mixed ANOVA for genotype, ns for interaction). Data are presented as mean ± SEM

Monitoring home cage activities allows for observations of basic behavioral characteristics reflecting various abilities, including sustained non-selective attention in mice. During a 1-week observation period, netrin-G1 KO and netrin-G2 KO mice exhibited normal circadian rhythms. Differential phenotypes (Fig. 5a, b), however, were observed in their mean horizontal and vertical activities over 6 days. Nocturnal mice show biphasic activity immediately after the lights-OFF and around the lights-ON. Netrin-G1 KO mice had hyper horizontal activity only in the first peak (Fig. 5a*left*). Their vertical activity decreased, particularly in the second peak (Fig. 5b*left*). In the late resting hours, before the lights-OFF, netrin-G1 KO mice were hyperactive in both horizontal and vertical activities, indicating abnormal sleep-wake patterns. In contrast, vertical activity was markedly decreased in netrin-G2 KO mice during both active and resting hours (Fig. 5b*bottom*), suggesting a marked deficit in non-selective attention.

To gain insight into the selective attention of netrin-G1 KO and netrin-G2 KO mice, we used the 5CSRTT [50]. Though not significant, netrin-G1 KO mice tended to make more omissions throughout spatial sessions 3–13. The number of omissions by netrin-G2 KO mice was indistinguishable from that of their WT control mice, (Fig. 5*c*). The number of erroneous pokes, reflecting inattention, was significantly increased in netrin-G2 KO mice throughout the testing sessions (Fig. 5*d right*). Because netrin-G1 KO mice tended to make omissions, we analyzed the erroneous poke rates per nose-poke response, which revealed increases in erroneous poke rates in netrin-G1 KO mice at later sessions (Fig. 5*e left*).

In addition to assessing visual-spatial attention capabilities, the 5CSRTT evaluates impulsive motor behavior in mice [50, 51]. Anterior cingulate, ventromedial prefrontal cortex and the ventral striatum are suggested to have a role in impulsive activity [52]. Between every trial, there is a short ITI wherein the animal must withhold all responses to identify the cue location, and premature responses during this interval are recorded as a measure of impulsivity. Both netrin-G1 KO and netrin-G2 KO mice exhibited tendencies toward impulsive behaviors during the test (Fig. 5*f*), although the tendency did not reach statistical significance in either case. A noteworthy difference in the dynamics of their phenotypes, however, was observed. Regardless of the variable duration of the ITI, premature responses exhibited by netrin-G1 KO mice occurred in the early sessions. The number of premature responses by netrin-G2 KO mice gradually increased in later sessions. These observations indicated that netrin-G1 and netrin-G2 are differentially involved in inhibitory control at different blocks. Netrin-G1 is responsible for impulsivity control through all the sessions, whereas netrin-G2 is responsible for impulsivity control under highly demanding conditions.

Thus, these findings suggest that netrin-G1 and netrin-G2 have crucial roles in distinct circuit mechanisms involved in attention and inhibitory control under different conditions.

### Netrin-G1 and Netrin-G2 differentially regulate postsynaptic properties

Netrin-Gs localize to presynaptic terminals and NGLs localize at the corresponding postsynaptic terminals [22, 25, 34]. Netrin-G1 and netrin-G2 differentially control synaptic plasticity in distinct pathways through the modulation of presynaptic properties [34]. There is little evidence, however, that netrin-Gs affect postsynaptic properties in vivo.

**Fig. 6:**
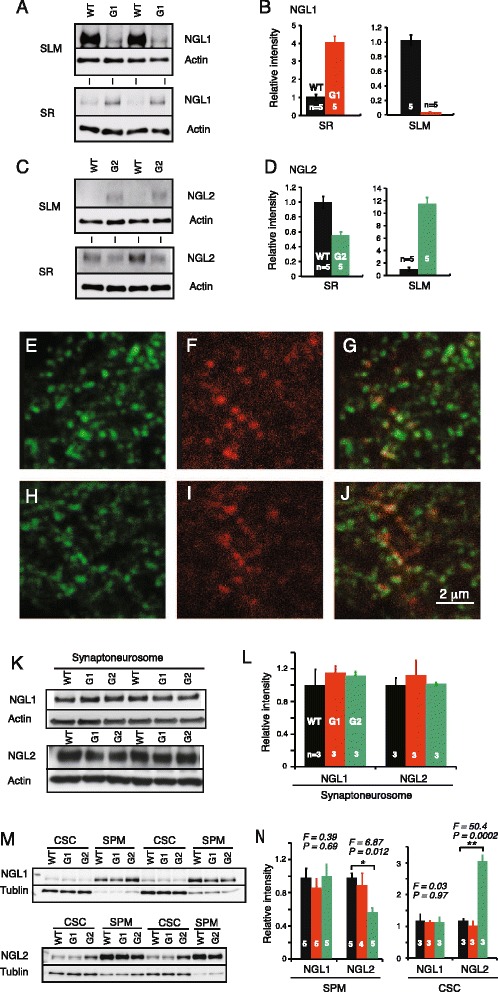
Postsynaptic changes in netrin-G KO mice. **a** and **b** Layer selective distribution of NGL1 in the hippocampal CA1 of netrin-G1 KO and WT mice. **a** Western blot images for NGL1 and actin in the SLM and SR samples. **b** Relative intensities of NGL1 normalized to actin (sample = animal numbers are indicated in the columns) indicate diffusion of NGL1 across layers of netrin-G1 KO mice. **c** and **d** Layer-specific distribution of NGL2 in the hippocampal CA1 of netrin-G2 KO and WT mice. **C**, Western blot images for NGL2 and actin in the SLM and SR samples. **D**, Relative intensities of NGL2 normalized to actin (sample = animal numbers are indicated in the columns) indicate diffusion of NGL2 across layers of netrin-G2 KO mice. **e**-**j** Confocal microscopy images obtained after dual immunohistochemistry for PSD-95 (green) and NGL-2 (red) in the cortex layer 4 of WT (**e**-**g**) and netrin-G2 KO (**h**-**j**) mice revealed that NGL-2 was co-localized with a postsynaptic marker in both WT and netrin-G2 KO mice (scale bar: 2 μm). **k** and **l** Western blot immunoassay for NGLs in synaptoneurosome fractions of WT, netrin-G1 KO, and netrin-G2 KO mice revealed no significant differences among genotypes (sample = animal numbers are indicated in the columns). **m** and **n** Western blot immunoassay for NGLs in CSC and SPM fractions of WT, netrin-G1 KO, and netrin-G2 KO mice. **N**: The relative intensities of NGLs normalized to β-tubulin were compared in SPM fractions (sample = animal numbers are indicated in the columns; 1-way ANOVA, * < 0.05 *post*-*hoc* Scheffe’s test). Although the sub-synaptic localization of NGL-1 was not altered in netrin-G1 KO mice, the sub-synaptic localization of NGL-2 was altered in netrin-G2 KO mice. The decrease in NGL-2 in the SPM fraction was associated with a marked increase in NGL-2 in the CSC fraction in netrin-G2 KO mice. Data are presented as mean ± SEM

We previously demonstrated that netrin-G deficiency disrupts the laminar-specific distribution of NGLs and a lack of netrin-G1 or netrin-G2 has no effect on the expression level of NGL1 and NGL2 [22]. Here, we further examined whether the synaptic and subcellular localization of NGLs is changed in netrin-G KO mice. First, we dissected the hippocampal CA1 layers of netrin-G1 KO, netrin-G2 KO, and their WT controls using a laser microdissecting system (Leica Microsystems) and examined the relative amounts of NGL1 and NGL2 by Western blot immunoassay (Fig. 6a-d). In WT mice, NGL1 and NGL2 preferentially distributed in the SLM and SR, respectively. In the netrin-G1 KO mice, however, the segregated distribution of NGL1 was abolished. Similarly, the segregated distribution of NGL2 was abolished in netrin-G2 KO mice. These findings together with immunohistochemical data [22] suggest lateral diffusion of NGL1 and NGL2 on the dendrites in the absence of presynaptic ligands. Confocal microscopic studies, however, revealed punctate NGL2 signals near postsynaptic marker PSD95 in the cerebra of netrin-G2 KO mice (Fig. 6e-j). To investigate the mechanisms underlying these observations, we performed a Western blot immunoassay for NGL1 and NGL2 in synaptoneurosome fractions [53] from the cerebra of netrin-G1 KO and netrin-G2 KO mice and their control mice (Fig. 6*k*, *l*). We detected comparable amounts of NGLs in the samples from all genotypes. The synaptoneurosome fraction comprises presynaptic sacs attached to postsynaptic sacs and contains the original content of the synaptic cytoplasm [53]. To examine whether the sub-synaptic localization of the NGLs changed, we used osmotic shock to break the sacs of synaptoneurosome fractions and further obtained SPM and CSC fractions. The purity of subcellular fractions was assessed by synaptic markers (data not shown). Western blotting for NGLs in these fractions revealed that NGL1 distributed largely within the SPM fraction in all genotypes (Fig. 6*m*). A substantial portion of NGL2, however, was detected in the CSC of netrin-G2 KO mice but not in that of WT mice (Fig. 6*m*). Quantitative analysis indicated that ~40 % of NGL2 in netrin-G2 KO mice trans-localized to the CSC compared with WT mice (Fig. 6*n*). These findings indicate that substantial amounts of NGL2 but not NGL1 are internalized at the postsynaptic sites in the absence of their ligands. Thus, these data suggest that netrin-G2 regulates the lateral translocation of NGL2 along the dendrites and the vertical translocation of NGL2 at the postsynaptic sites, and that netrin-G1 primarily regulates the lateral translocation of NGL1 along the dendrites. These different mechanisms might underlie differences in the regulation of synaptic transmission in netrin-G1- and netrin-G2-dependent circuits.

**Fig. 7:**
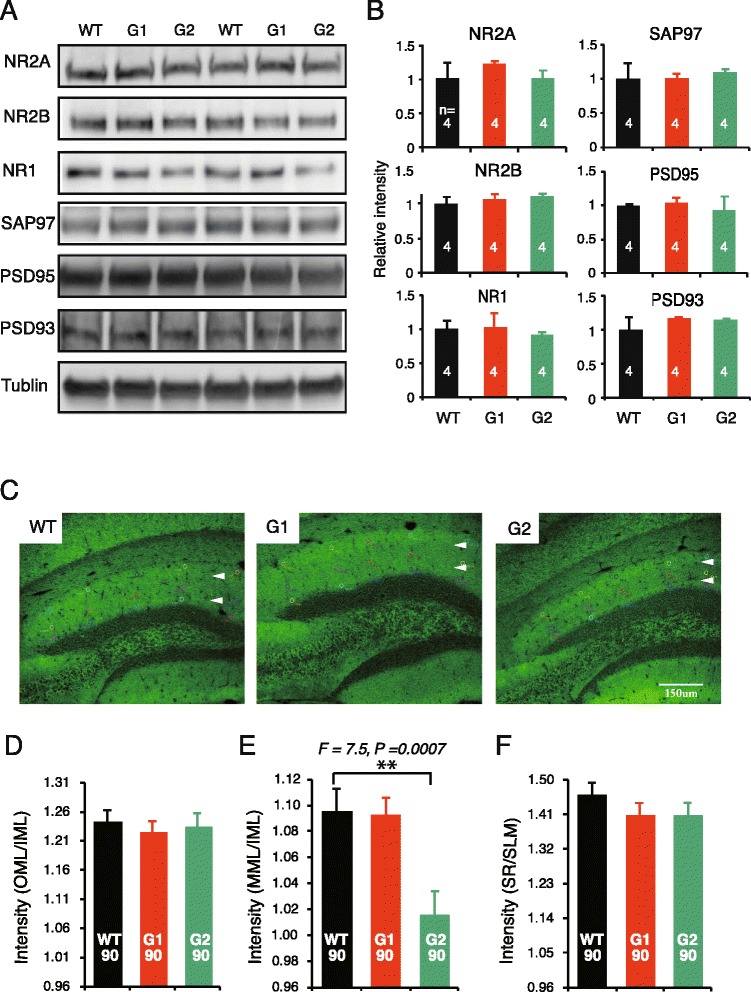
Altered distribution of PSD-95 in netrin-G2 KO mice. **a** and **b** Western blot analyses for postsynaptic molecules in the cerebral samples. There were no detectable changes in the amounts of molecules examined (sample = animal numbers are indicated in the columns). **c** Immunohistochemical staining for PSD-95 in the dentate gyrus of WT, netrin-G1 KO, and netrin-G2 KO mice. White arrowheads indicate approximate borders among OML, MML, and IML. The difference in fluorescence intensity between MML and IML was much more distinct in netrin-G2 KO mice compared to WT and netrin-G1 KO mice (scale bar: 150 μm). **d** and **e** Relative intensities of PSD-95 in each layer were first calibrated by MAP-2 intensity, and then the ratio of the V_OML_/V_IML_, V_MML_/V_IML_, and V_SR_/V_SL_ values were calculated for further normalization. The relative intensity of PSD-95 was selectively decreased in the MML of netrin-G2 KO mice (1-way ANOVA, **P < 0.01 *post*-*hoc* Scheffe’s test). Intensities were measured from 5 ROI/layer/slice, 6 slices/animal, and 3 mice for each genotype. Total numbers of ROIs are indicated in the columns. IML, inner molecular layer; MML, middle molecular layer; OML, outer molecular layer; SL, stratum lacunosum-moleculare; SR, stratum radiatum. Data are presented as mean ± SEM

Next, we tried to explore the possible impact of the translocation of NGL at the postsynaptic compartment. Our yeast-two-hybridization screening indicated that both NGL1 and NGL2 bound all four Discs large homolog (Dlg) family members through their intracellular PDZ binding domain (data not shown). NGL2 reportedly precipitates PSD95, NR1, and NR2B from brain tissue [25]. We examined Dlg molecules and NR1, NR2A, and NR2B in the SPM by Western blotting immunoassay. No quantitative differences in these molecules were detected among the cerebral samples from netrin-G1/2 KO and WT mice (Fig. 7*a*, *b*). We also performed immunohistochemistry in brain sections for PSD95, PSD93, SAP102, SAP97, and NR1 to examine whether the expression patterns of these molecules exhibited any layer-specific changes in the hippocampus. Only in PSD95-stained samples was there an apparent difference among genotypes– the fluorescence signal in the medial molecular layer (MML) in netrin-G2 KO mice was distinct from that of the inner molecular layer (IML), while the difference between the MML and IML was rather mild in both WT and netrin-G1 KO mice (Fig. 7*c*). A semi-quantitative analysis of the PSD-95 signal intensity in the MML was then performed and normalized by MAP-2 signal intensity. Because neither netrin-G1 nor netrin-G2 distribute in the IML of WT mice, the results were further calibrated by the value of the IML. The V_MML/IML_ of netrin-G2 KO mice was significantly decreased compared with WT mice (Fig. 7*d*-*f*). No detectable change in PSD-95 intensity was detected in netrin-G1 KO mice or in other layers in netrin-G2 KO mice. This pathway-specific PSD95 alteration supports the notion that postsynaptic mechanisms have a role in the differential behavioral phenotypes of netrin-G1 KO and netrin-G2 KO mice.

## Discussion

### Division of labor for netrin-G1 and netrin-G2 in behavioral regulation

Our findings provide evidence that paralogs of the netrin-G subfamily have differential roles in various behavioral domains (Table 1). For the complex cognitive tasks, netrin-G1 KO mice had deficits in encoding spatial reference information and in the learning phase of the spatial working memory task. Netrin-G2 KO mice exhibited both impaired spatial reference memory and impaired working memory. In addition, netrin-G2 KO mice performed poorly in the procedural learning phase and attention phase of the 5CSRTT. Notably, although both KO mice exhibited deficits in attention, the deficit of netrin-G1 KO mice was associated with omission errors and that of netrin-G2 KO mice was characterized by erroneous pokes, supporting the diversification of behavioral function within a single task. Anxiety and fear are two highly related facets of emotion. The fear-conditioning task was solely influenced by netrin-G1, while anxiety appeared to be regulated by both genes. For anxiety evaluation tasks, it is especially interesting that netrin-G1 KO mice demonstrated reduced anxiety in the EPM test, but not the OF test, whereas netrin-G2 KO mice exhibited abnormalities in both tests. Evidently, evolution of this subfamily of the UNC6/netrin family endowed vertebrates with a more specialized and complex regulatory system, which in turn allows higher organisms to detect and discriminate different contexts and produce more specific responses and actions. Previously, we and others revealed differential expression patterns of netrin-G paralogs in distinct neuronal circuits [18, 19, 33] and its significance in circuit specification, even in a single cell [22, 34]. Evolutional acquisition of differential transcriptional activities of paralogs is an apparently efficient strategy for enlarging the behavioral repertoire of vertebrates to increase their adaptive ability to survive in complex changing environments. In summary, netrin-G1 and netrin-G2 genetically dissect different behaviors and even different details of the same behavioral paradigm. Considering expression patterns and behavioral phenotypes, we suggest that netrin-G2 has a crucial role in both ends of bottom-up and top-down circuits and netrin-G1 is crucial in proper processing of bottom-up signals. Definitive functioning sites of these molecules underlying specific behavioral outputs remained to be determined in future studies.

**Table 1:** Summary of behavioral phenotypes of netrin-G1 KO and netrin-G2 KO mice

Classification			Test	netrin-G1 KO	Cohort/Sequence	netrin-G2 KO	Cohort/Sequence	Source
Basic condition and sensory-motor ability	Basic condition		Appearance	light body weight		normal		Nishimura-Akiyoshi et al., 2007
			Reflex (righting, posture, eyeblink, ear twitch, whisker orienting)	normal	1G/1	normal	2 F/2	data not shown
	Sensory ability		Optometry	normal (enhanced contrast sensitivity)	1B	reduced acuity	2I	Fig. 2a, b
			Visual cliff	normal	1G/2	deficits in depth perception	2 F/4	Fig. 2c
			ABR	normal	1 F/3	deficits	2E/2	Fig. 2d, e
			Acoustic startle response	normal	1 F/2	deficits	2E/1	Fig. 2f
			Foot shock sensitivity	normal	1A/4	normal	2A/3	Fig. 2g
	Motor ability	Low-demand	Runway	normal	1G/1	normal	2 F/3	DNS
			Grip strength	normal	1G/1	normal	2 F/3	DNS
		High-demand	Rotarod	normal	1 F/1	deficits (motor coordination or motivation)	2 F/1	Fig. 2h
			Hanging wire	normal	1G/1	deficits (motor coordination or motivation)	2 F/2	Fig. 2i
Emotion			Open field	normal	1D/1	low anxiety	2C/1	Fig. 3a, b
			Elevated plus maze	low anxiety, impulsive behavior	1 J	low anxiety	K/1	Fig. 3c
			Social interaction	enhanced interaction to novel mice	1D/3	enhanced interaction to novel and familiar mice	2C/3	Fig. 3d
			Fear conditioning	attenuated fear response	1A/2	normal	2A/2	Fig. 3e
Cognition	Simple-form		Nobel object recognition	normal	1D/2	normal	2C/2	Fig. 4a, b
			Y maze	normal	1I/3	hyper activity	2 J/2	Fig. 4c, d
	Complex-form		Morris water maze (hidden)	deficits in goal-directing	1 K	deficits in spatial learning and memory	2A/1	Fig. 4e-g
			Morris water maze (visible)	deficits in goal-directing	1 K	normal	2A/1	Fig. 4h
			Radial maze	slow learning, impulsive behavior	1A/1	working memory deficits	2C/4	Fig. 4i
			5CSRTT (learning stage)	normal	1I/2	slow procedural learning	2 J/1	Fig. 4j
	Attention		Home cage	abnormal sleep, modest decline in non-selective attention	1I/1	decline in non-selective attention	2G	Fig. 5a, b
			5CSRTT	attention deficit (mainly associated with omission error)	1I/3	attention deficit (mainly associated with erronious poking)	2 J/2	Fig. 5c-d
				impulsive tendency	1I/3	impulsive tendency at high-demanding condition	2 J/2	Fig.5f

If same cohorts were subjected for multiple tests, resting periods longer than several days were applied between tests. (1D/1, 2, and 3), (2A/1, 2, and 3), (2C/1, 2, and 3) were carried out in consequtive days

### Division of labor for netrin-G1 and netrin-G2 in synaptic properties

Our study also addressed the question of whether netrin-G isoforms genetically diversify synaptic properties. We previously reported that in acute hippocampal slices, post-tetanic potentiation and LTP were attenuated in the SLM of netrin-G1 KO mice and augmented in the SR of netrin-G2 KO mice due to presynaptic mechanisms [34]. Netrin-G1 and netrin-G2 interact with postsynaptic NGL1 and NGL2, respectively, at the synaptic cleft. Our findings in this study further demonstrate that they can also segregate synaptic properties on the postsynaptic side. The decreased PSD95 intensity was only detected in the MML of netrin-G2 KO mice. None of the Dlg molecules showed intensity changes in netrin-G1 KO mice. It is noteworthy that when AMPA receptor-mediated fast EPSPs are recorded in the hippocampal SR, SLM, MML, and outer molecular layer of either netrin-G1 KO or netrin-G2 KO mice upon stimulation of the Schaffer collateral, temporoammonic, medial perforant, and lateral perforant pathways, respectively, relative postsynaptic responsiveness to presynaptic activity is significantly altered only in the MML of netrin-G2 KO mice, which might be associated with a reduction in the PSD95 intensity. The C-terminal domain of NGL2 interacts with PSD95 [25]. CDKL-5 binds and phosphorylates NGLs, and stabilizes the NGL-PSD-95 association [54]. It was recently reported that Ca^2+^ flow initiated by neuronal activity can disrupt the association between CDKL-5 and PSD-95 [55]. It is feasible that netrin-G/NGL/CDKL-5-dependent signaling cascades play a role in regulating synaptic plasticity under some circumstances. One important characteristic of the synapse is that biochemical mechanisms are often confined to “microdomains”, and the resulting synaptic plasticity affects only a specific synapse or a sub-domain within a synapse [56, 57]. The circuit-specific nature of the netrin-Gs/NGLs interaction within a single neuron [22, 34] may be a mechanism of the microdomain-specific synaptic plasticity. The findings of this study provide evidence that NGL2 but not NGL1 is internalized near the synapses in the absence of the presynaptic ligand. The differential mechanisms underlying sub-synaptic trans-localization of NGL1 and NGL2 may work in an activity-dependent manner and contribute to diversifying synaptic functions in distinct circuits.

### Netrin-G1 and netrin-G2 diversify vertebrate behaviors through differential regulation of synaptic properties on distinct neural circuits

Seth Grant’s group observed a marked change in the signaling complexity at the invertebrate-vertebrate boundary, with an expansion of key synaptic components, including receptors, scaffold proteins, and adhesion proteins. They propose that the increase in molecular signaling complexity contributes to the increased capacity for behavioral complexity in vertebrates [58]. Indeed, mutants of two distinct mammalian NR2 subunits (NR2A and NR2B) have distinct synaptic and behavioral phenotypes [16, 59–64]. A similar rule applies to the four paralogs of the Dlg family (SAP-97, PSD-93, SAP-102, and PSD-95) [15, 65, 66]. Our current findings provide support for this concept. Grant and his colleagues also suggest that vertebrate-specific synaptic molecules preferentially contribute to brain regional specialization in evolution. The findings of the netrin-G subfamily reported here indicate that synaptic molecular evolution further contributes to functional diversities at the circuit level, even in the same brain region. Another pair of vertebrate-specific presynaptic molecules well known for their complementary expression in the central nervous system is vesicular glutamate transporter 1 and 2 (*Vglut1* and *Vglut2*). These transporters functionally categorize synapses with low and high glutamate basal release probabilities in different neural pathways [67–69]. Netrin-G1 and netrin-G2, through their ligands, may have advantages to bi-directionally balance the activity-dependent plasticity from both pre- and postsynaptic sides.

## Conclusions

The findings of the present study together with those of previous studies demonstrate that two vertebrate-specific GPI-linked synaptic adhesion molecules, netrin-G1 and netrin-G2, crucially differentiate synaptic properties from both presynaptic and postsynaptic sites. This property confers specific physiologic properties to complementary neural pathways, and thus differentially modulate the behavioral repertoire of vertebrates at a fine-scale level. Further experiments with netrin-G1 and netrin-G2 conditional KO mice combined with other genetic tools will help to precisely and definitively dissect the specific neural circuits underlying behavioral phenotypes.

## Methods

### Animals

All experimental procedures were performed in accordance with the guidelines of the RIKEN Institutional Animal Care and Experimentation Committee. Netrin-G1 (*Ntng1*) KO and netrin-G2 (*Ntng2*) KO mouse strains [22] were maintained as C57BL6J congenic heterozygotes. Homozygous KO and wild-type (WT) control littermates were obtained by crossing the heterozygotes, and male mice (2 – 9 mo-old) were used for behavioral studies. The netrin-G1 KO mice carry NLS-LacZ [22]. A nertin-G2 KO mouse line carrying NLS-LacZ was reported previously [33]. Using the similar strategies, we here generated additional knockin mouse lines carrying Tau-EGFP and Tau-mCherry at netrin-G1 and netrin-G2 gene loci, respectively.

### Antibodies (Abs)

Primary Abs used in this study included rabbit polyclonal Abs for NGL1 and NGL2 [22], mouse monoclonal anti-PSD95 (ABR), mouse monoclonal anti-PSD95 (BD Transduction Laboratories), rabbit polyclonal anti-PSD93 (Synaptic System), mouse monoclonal anti-SAP102 (Stressgen), mouse monoclonal anti-SAP97 (Stressgen), rabbit polyclonal anti-NR1 (Chemicon), mouse monoclonal anti-NR2A (Chemicon), mouse monoclonal anti-NR2B (Chemicon), rabbit polyclonal anti-MAP2 (Millipore), mouse monoclonal anti-Actin (Millipore), and mouse monoclonal anti β-tubulin III (Abcam). The secondary antibodies used for immunofluorescence were Alexa Fluor 488, and 546 donkey anti-mouse or rabbit IgG (Molecular Probes). The horseradish peroxidase-conjugated secondary antibodies used for Western blot analysis were from GE Healthcare and Jackson Laboratories.

### Behavioral analyses

Mice were group-housed by combining each genotype (4 mice/cage) and maintained under a 12 h light–dark cycle (lights on 8:00 AM, lights off 8:00 PM). Behavioral tests were conducted during the light phase except for monitoring home cage activities. All tests were carried out in a genotype-blind manner. Cohorts used and sequences of tests are indicated in Table 1.

1. Neurologic reflexes: Righting, posture, eyeblink, ear twitch, and whisker orienting reflexes were examined as previously described [70].
2. Optometry: Visual acuity and contrast sensitivity were examined using a virtual optokinetic system (OptoMotry; CerebralMechanics Inc.) according to the procedure described previously [71]. Each mouse was placed on a central elevated platform surrounded by display monitors. Head and neck movements elicited by rotating visual stimuli presented on display monitors were recorded.
3. Visual cliff test: This test was conducted in a box (50 × 50 × 50 cm), according to the procedure described previously [72] with a minor modification. The test evaluates visual depth perception and anxiety of animals. A transparent Plexiglas board (50 × 50 cm), which was positioned 50 cm above the bottom of the box, had two distinctive regions: the platform and drop-off regions. The bottom of the box and the platform region were covered with black and white checkerboard (2 × 2 cm each) contact paper, whereas the drop-off region had no contact paper, giving the visual appearance of a cliff. A long bar (5 cm W × 3 cm H × 50 cm L) was placed to divide the platform and drop-off regions. A mouse was placed in the platform region, and its locomotor activity was monitored for 5 min. The distance traveled and percent time spent in the specific regions were observed.
4. Auditory brainstem response (ABR): ABR was recorded as described previously [73]. Electrodes were placed at the vertex (ground) and ventrolateral to the left (reference) and right ears (active). ABR was measured using the waveform storage and stimulus control functions of Scope software (PowerLab2/20; ADInstruments), and EEG recording with an extracellular amplifier (AC PreAmplifier, P-55, Astro-Med Inc.). The unilateral tone stimulus produced by a speaker (ES1 spc; BioResearch Center Nagoya, Japan) was fed into the auditory canal of the right ear. ABR waveforms were bandpass-filtered (<30 and >3000 Hz), amplified 100,000 times, and recorded for 12.8 ms at a sampling rate of 40,000Hz. Two hundred-fifty recordings were averaged.
5. Acoustic startle response: A startle reflex measurement system (MED Associates) was used. Each mouse was placed in a Plexiglas cylinder, and set in a chamber with background noise (65 dB white noise). After a 5-min acclimation period, auditory stimuli with varied intensities (9 levels, 70 ~ 120 dB white noise, 40 ms duration) were presented in a quasi-random order at random inter-trial intervals (ITI, 10–20 s). Acoustic startle response is mediated by neural circuits in the lower brain stem, including the central nucleus of the pontine reticular formation [74].
6. Rotarod test: Motor coordination was examined with a constant-speed (20 revolutions per minute) rotarod (Model 7600, UGO BASILE) for 3 days, as described previously [75]. Mice were given three trials per day, and the testing was terminated upon their fall or upon reaching the maximum duration (5 min). Latency to fall from the rotarod was used as the dependent variable of performance.
7. Elevated runway task: Motor coordination was examined by this task as described previously [76]. A wooden runway (110 cm L, 2 cm W) was elevated at one end to create a 30° angle between the ends. At the higher end, an escape platform was placed to allow mice to leave the narrow runway. The lower end was 22 cm above the ground to deter the mice from getting off the apparatus. Low obstacles were placed to impede the progress of the mice at 6-cm intervals along the runway. Initially, the mice were placed on the escape platform for 30 s. Next, the mice were placed on the lower end of the elevated runway and allowed to move freely on the runway. The latency to reach the escape platform was recorded for four consecutive trials. Maximum duration per trial was set to 120 s.
8. Neuromuscular strength test: The hanging wire test was conducted to determine neuromuscular strength in experimental mice [70]. Each mouse was placed on a wire cage lid and the lid turned upside down, approximately 20 cm above soft bedding material. Latency to fall from the lid to the soft bedding was recorded, with a 60-s cut-off time.
9. Home cage activity: Locomotor activity was measured for 7 days as described previously [77]. Each mouse was single-housed in a transparent cage (20.7 cm [W] × 36.5 cm [L] × 14 cm [H]) containing bedding, and vertical and horizontal movements were detected using infrared area sensors (Scanet; Melquest, Toyama, Japan). The mice were maintained under a 12-h light and dark cycle (lights on: 8:00–20:00) with food and water available ad libitum. The experiment was started at approximately 11:30 am. All the data on the locomotor activity were obtained by averaging the levels of activity during each hour. The data for Day 1 light phase (11:30 am to 20:00 pm) were excluded from the analysis.
10. Open field and novel object recognition tests: Open field test is used to evaluate the mice for their motor ability and anxiety level for new environment, and novel object recognition is used for assessing learning and memory and is reflected by conflicts between curiosity and anxiety of animals [78]. These tests were conducted continuously for 3 days. On Day 1, each mouse was placed in the center of the open field apparatus (50 cm [L] × 50 cm [W] × 40 cm [H]) illuminated at 70 lux (surface level of the arena). Horizontal locomotor activity and time spent in the center area were monitored for 25 min with a CCD camera and processed with NIH Image OF software (O’Hara & Co.). On Day 2, each mouse was placed in the same apparatus in which two identical objects, either cones or hexagon pillars, were positioned equidistant from each other and the corners of the cage. Animal behavior was recorded for 10 min. On Day 3, one of the familiar objects was replaced with a novel object. The animal behavior was again recorded for 10 min. The percentage of time spent exploring and the number of contacts of mice with the objects were measured.
11. Elevated plus maze test: This apparatus consisted of two open arms (8 cm × 25 cm) and two closed arms of the same size with 15-cm high transparent walls. The maze was elevated 50 cm above the floor and illuminated at 200 lux (surface level of the maze). Each mouse was placed on the central platform of the maze, and exploratory behavior was monitored with a CCD camera for 10 min and analyzed with the NIH Image EP software (O’Hara & Co.). Elevated plus maze is widely used to evaluate anxiety-related behaviors in rodents [79].
12. Social interaction test: Adult test mice were habituated to individual cages (28.5 cm [L] × 17.5 cm [W] × 12 cm [H]) for 15 min. A novel juvenile male (C57BL/6 J, 3–4 weeks of age) was placed into the cage, and their interaction was observed for 2 min. Twenty-four hours later, the same procedure with the same pair of mice was repeated. The duration of their interaction was recorded using a hand-held stopwatch, and the nature of the behavior was carefully noted by a trained observer. The test was conducted to evaluate social memory of mice.
13. Forced swim test: This rest provides a paradigm for evaluating depression-related behaviors in rodents [80]. Each mouse was placed in a cylinder (25 cm H, 20 cm diameter) filled with 15 cm-deep water (temperature 25 ± 1 °C) for 6 min. After 24 h, the same procedure was repeated. Floating time (defined as the lack of swimming and/or minimal movement of one leg, sufficient to keep the head above the water) was recorded.
14. 5-choice serial reaction time task (5-CSRTT): The 5-CSRTT was performed according to the procedure described previously [51] with minor modifications for evaluating attention and impulsive behaviors [50]. In brief, mice were handled and food restricted to reduce their body weight to approximately 85 % of free-feeding weight. Mice were habituated to the 5-CSRTT chamber (O’Hara & Co.) for 2 days, trained to consume 10-mg sugar pellets (TestDiet) from a food dispenser for 3 days, and trained to associate nose-pokes with food in a non-spatial manner for 3 days (100 trials/day). In the following spatial stages, only 1 of 5 holes was illuminated in a random manner with a different ITI and a limited hold (LH) of the green signal light behind of the hole: spatial stage 1 (ITI 2 s, LH 60 s), stage 2 (ITI 10 s, LH 60 s), stage 3 (ITI 10 s, LH 30 s), stage 4 (ITI 10 s, LH 10 s), stage 5 (ITI 15 s, LH 30 s), stage 6 (ITI 20 s, LH 10 s), stage 7 (ITI 20 s, LH 5 s), stage 8 (ITI 20 s, LH 2 s), stage 9 (ITI 20 s, LH 1 s), and stage 10 (ITI 20 s, LH 0.8 s). At stages 11 to 13, the ITI was stepped down to 15 s, 10 s, and 5 s. In spatial stages 2–13, the house light was extinguished for 2 s if a mouse did not perform a nose-poke within the LH (omission) or performed a nose-poke into a wrong hole (incorrect). A nose-poke before the ON signal light was considered a premature nose-poke.
15. Y-maze spontaneous alteration test: The Y-maze task was performed essentially as described previously [81]. Spontaneous alteration provides a simple paradigm for assessing working memory [81, 82]. Each arm was 40 cm long, 12 cm high, 3 cm wide at the bottom, and 10 cm wide at the top. The maze was illuminated at 70 lux (surface level of the maze). Each mouse was placed at the end of a start arm, and was allowed to move freely through the maze during a 5-min observation period. An alternation was defined as entry into all three arms without repetition on consecutive choices. The percent of alternation was calculated as (actual alternations/maximum possible alternations) × 100.
16. Morris water maze test: The Morris water maze test was performed according to the procedure described previously [83]. The test is widely used for evaluating hippocampus-dependent spatial reference memory [43]. The water pool, 1 m in diameter, was illuminated with 250 lux white fluorescent light at the maze-surface level. Mice were given four trials per day in a spaced manner for 7 consecutive days for a hidden-platform paradigm. A 60-s probe test was performed on Day 8. A visible-platform paradigm was carried out for 3 consecutive days (6 trials per day).
17. Fear conditioning test: Contextual and cued fear conditioning tests were performed according to the procedure described previously [83]. Hippocampus, amygdala and periaqueductal gray areas are differentially involved in these paradigms [42, 84]. Contextual and cued tests were performed at 24 and 48 h after the conditioning, respectively. Using the same conditioning chamber, foot shock sensitivity was assessed by giving mice electrical shocks of increasing intensity, ranging from 0.05 mA to 1 mA in 0.05-mA steps and monitoring their behavior (i.e., flinch, vocalization, and jump), as described previously [85].
18. 8-arm radial maze test: The test evaluates spatial working memory [45] and requires hippocampus, prefrontal cortex and striatal circuits [86]. Each arm (8 × 40 cm) radiated from an octagonal central platform (28 cm diameter) of the maze (O’Hara & Co.). Arms were surrounded by transparent walls (10 cm or 24.8 cm high). The low and high wall mazes were used for netrin-G2 KO mice and netrin-G1 KO mice, respectively. The maze was illuminated with 70 lux white fluorescent light at the maze-surface level. Five days prior to commencement of the experiment, mice were food deprived while water was provided ad libitum to approximately 85 % of the free-feeding weight. Each trial was initiated by placing an individual mouse on the octagonal central platform with all guillotine doors to the arms closed. After 5 s, all doors were opened, and the mouse was allowed to explore the maze freely and identify a non-odorous 20-mg sugar pellet (TestDiet) located at the end of each arm. The opening and closing of the arms was controlled by software. After the animal consumed a sugar pellet and returned to the center, all the doors were closed for 5 s and re-opened simultaneously to allow the animal to search for the remaining food pellets. Once all eight food pellets were consumed, or the maximum duration of 900 s elapsed, the animal was returned to its home cage. The animal was given one trial per day and trained for 14 days. The number of total entries, the number of correct choices in the first eight entries, number of reentry errors, and duration of time required to consume all the pellets were recorded for each trial. Re-entrance of a previously visited arm was counted as a working memory error. Movement of each mouse was monitored with a CCD camera and analyzed with the NIH Image RM software (O’Hara & Co.). Infrared beam sensors detected sugar pellet consumption.

### Subcellular fractionation

All steps were performed at 4 °C. Synaptoneurosome purification was performed according to the previously published protocol [53]. Briefly, homogenized cerebrum tissue was sequentially passed through 100-μm, 41-μm, and 5-μm filters. After centrifugation and washing, the pellet was set aside for the next step. Crude membrane protein (Crude synaptosome fraction) extraction was performed according to the online protocol from Michael Ehlers (http://sici.umh.es/docs/Brain_membrane_synaptic_fractions_IPO.pdf) with minor modifications. Briefly, homogenized cerebrum was centrifuged at 1400 g for 15 min and the supernatant was further centrifuged at 12,000 g for 40 min, and then the washed pellet was set aside for the next step. Synaptic plasma membrane (SPM) and condensed synaptic cytoplasm (CSC) fractions were obtained. Briefly, the synaptoneurosome or crude synaptosome fraction pellet were lysed by hypoosmotic shock in ice cold water and centrifuged at 25,000 g for 40 min to yield a supernatant (SC, crude synaptic cytoplasm fraction) and a pellet (P3, lysed synaptosomal membrane fraction). P3 was resuspended in HEPES buffered sucrose (0.32 M sucrose, 5 mM HEPES pH = 7.4), layered on top of a discontinuous gradient containing 0.8 to 1.0 to 1.2 M sucrose, and centrifuged at 150,000 g for 2 h. The SPM was recovered in the layer between 1.0 and 1.2 M sucrose. The CSC was obtained by centrifugation of the SC at 165,000 g for 2 h. All samples were quantified by BCA protein assay and then subjected to SDS-PAGE, transferred to PVDF membrane (BioRad), and Western blot analyses was performed with antibodies against the proteins listed above.

### Laser microscopic dissection

Brains were removed and frozen immediately after exsanguination by perfusing with saline. Serial coronal sections (20 μm) were collected on Frame Slides (POL-membrane, 0.9 μm, Leica Microsystems) throughout the hippocampus. The sections were stained with 0.005 % toluidine blue, dried, and stored at −80 °C until use. Sampling of hippocampal CA1 subregions, the stratum lacunosum moleculare (SLM) and stratum radiatum (SR), was performed by using a laser microdissection microscope (AS LMD, Leica Microsystems). The SLM and SR were precisely dissected from 60 sections per mouse. The samples from one side of the hippocampus were pooled and lysed directly in 60 μl of modified SDS-PAGE sample buffer (2 M urea, 5 % SDS, 62.5 mM Tris [pH6.8], 10 % glycerol, 5 % 2-mercaptoethanol, 0.005 % bromophenol blue). The lysate was completely homogenized by sonication, centrifuged at 8500 g for 5 min, and the supernatant (8 μl/lane) was subjected to Western blot analysis for NGL1 and NGL2.

### Immunohistochemistry and semi-quantitative analysis

For PSD95 fluorescence intensity analysis, three animals/genotype were perfused with 4 % paraformaldehyde, dehydrated, embedded into paraffin, and sectioned. Six coronal sections from each animal were immunostained and analyzed. Paraffin-embedded sections (5 μm thickness) were deparaffinized, rehydrated, and processed for heat-induced epitope retrieval. Blocking with 5 % NGS/0.3 % Triton X-100 was done for 1 h at room temperature. The slices were then incubated with primary antibodies for 12 h at 4 °C and fluorescence-conjugated second antibodies for 4 h at room temperature. Images were captured using a confocal microscope (Leica TCS SL) with a sequential scanning mode. All analyses for the images were performed blind. Five evenly distributed verticals to the hippocampus curve’s tangent were defined on each coronal section and the circular areas (100 μm^2^) from each layer localized on the same vertical plane were analyzed. The root mean square value of both PSD95 (green) and MAP2 (red) were measured (using the defaulted software of Leica TCS SL confocal microscope) from a total of 1350 ROIs (90 ROIs for each layer/genotype). The PSD95 value was calibrated by RMSPSD95/RMSMAP2. The ratio of Vouter/Vinner, Vmiddle/Vinner, and Vradiatum/Vlacunosum from the ROI localized on the same vertical line were calculated and compared among genotypes. Western blots were quantified using ImageJ software and the plot values were normalized by the ratio to actin or β-tubulin III.

### Statistics

The data were analyzed using a one-way ANOVA, two-way ANOVA, and mixed ANOVA, and Student’s two-tailed *t* test with IBM SPSS Statistics (ver. 21). All values are expressed as mean ± SEM. P values less than 0.05 were considered significant.

#### Ethics approval and consent to participate

All experimental procedures were performed in accordance with the guidelines of the RIKEN Institutional Animal Care and Experimentation Committee.

#### Consent for publication

All authors agree to publish the work in Molecular Brain.

#### Availability of data and material

All data and materials are available upon requests.

## Abbreviations

GPI: Glycosylphosphatidylinositol
KO: Knockout
WT: Wild-type
ABR: Auditory brainstem response
5-CSRTT: 5-choice serial reaction time task
ITI: Inter trial interval
LH: Limited hold
SPM: Synaptic plasma membrane
CSC: Condensed synaptic cytoplasm
SLM: Stratum, lacunosum moleculare
SR: Stratum radiatum
ROIs: Regions of interest
